# Bis(benzyl­ammonium) di­hydrogen diphosphate

**DOI:** 10.1107/S1600536813032455

**Published:** 2013-12-04

**Authors:** Ahlem Ben Saad, Adel Elboulali, Nicolas Ratel-Ramond, Rzaigui Mohamed, Samah Akriche Toumi

**Affiliations:** a Laboratoire de Chimie des Matériaux, Faculté des Sciences de Bizerte, 7021 Zarzouna Bizerte, Tunisia; b CEMES-CNRS, 29 rue Jeanne Marvig, 31055 Toulouse cedex 4, France

## Abstract

The asymmetric unit of the title salt, 2C_6_H_5_CH_2_NH_3_^+^·H_2_P_2_O_7_^2−^, contains two independent benzyl­ammonium cations and a di­hydrogen diphosphate dianion. In the crystal, O—H⋯O and N—H⋯O hydrogen bonds link the cations and anions, forming a two-dimensional network parallel to (010). Within this network, weak C—H⋯O hydrogen bonds are observed.

## Related literature   

For the chemistry of diphosphate materials, see: Ernester (1992 ▶); Lipscomb & Strater (1996 ▶); Centi *et al.* (1988 ▶); Chen & Munson (2002 ▶); Ballarini *et al.* (2006 ▶). For details of hydrogen bonds, see: Desiraju (1991 ▶); Steiner (2002 ▶). For related structures, see: Akriche & Rzaigui (2005 ▶, 2008 ▶); Ahmed *et al.* (2006 ▶); Elboulali *et al.* (2013 ▶).
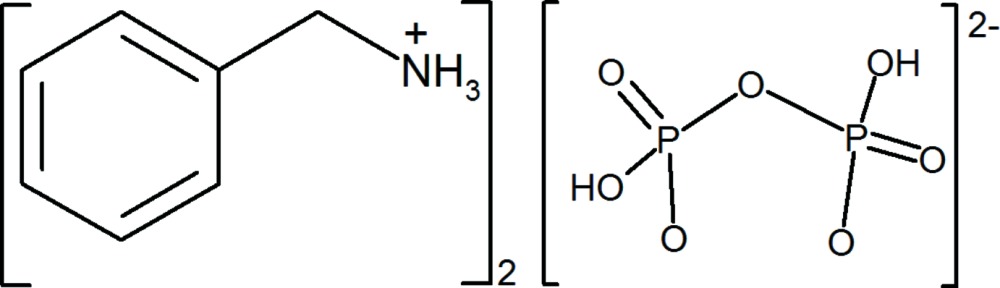


## Experimental   

### 

#### Crystal data  

2C_7_H_10_N^+^·H_2_P_2_O_7_^2−^
*M* *_r_* = 392.27
Monoclinic, 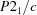

*a* = 8.1337 (2) Å
*b* = 28.9015 (9) Å
*c* = 8.4727 (2) Å
β = 113.449 (1)°
*V* = 1827.24 (9) Å^3^
*Z* = 4
Mo *K*α radiation
μ = 0.28 mm^−1^
*T* = 293 K
0.3 × 0.2 × 0.1 mm

#### Data collection  

Nonius KappaCCD diffractometer
27919 measured reflections
7410 independent reflections
5946 reflections with *I* > 2σ(*I*)
*R*_int_ = 0.025

#### Refinement  

*R*[*F* ^2^ > 2σ(*F* ^2^)] = 0.043
*wR*(*F* ^2^) = 0.127
*S* = 1.06
7410 reflections
230 parameters
H-atom parameters constrained
Δρ_max_ = 0.52 e Å^−3^
Δρ_min_ = −0.28 e Å^−3^

### 

Data collection: *COLLECT* (Hooft, 1998 ▶); cell refinement: *DIRAX/LSQ* (Duisenberg *et al.*, 2000 ▶); data reduction: *EVALCCD* (Duisenberg *et al.*, 2003 ▶); program(s) used to solve structure: *SHELXS97* (Sheldrick, 2008 ▶); program(s) used to refine structure: *SHELXL97* (Sheldrick, 2008 ▶); molecular graphics: *ORTEP-3 for Windows* (Farrugia, 2012 ▶) and *DIAMOND* (Brandenburg & Putz, 2005 ▶); software used to prepare material for publication: *WinGX* publication routines (Farrugia, 2012 ▶).

## Supplementary Material

Crystal structure: contains datablock(s) I. DOI: 10.1107/S1600536813032455/lh5670sup1.cif

Structure factors: contains datablock(s) I. DOI: 10.1107/S1600536813032455/lh5670Isup2.hkl

Supporting information file. DOI: 10.1107/S1600536813032455/lh5670Isup3.cml

Additional supporting information:  crystallographic information; 3D view; checkCIF report

## Figures and Tables

**Table 1:** Hydrogen-bond geometry (Å, °)

*D*—H⋯*A*	*D*—H	H⋯*A*	*D*⋯*A*	*D*—H⋯*A*
O1—H1*O*1⋯O6^i^	0.82	1.90	2.7208 (13)	174
O5—H5*O*5⋯O3^ii^	0.82	1.83	2.6061 (13)	158
N1—H1*N*1⋯O2	0.89	2.07	2.9292 (13)	162
N1—H2*N*1⋯O6^iii^	0.89	2.10	2.9698 (15)	166
N1—H2*N*1⋯O4^iii^	0.89	2.53	3.1493 (13)	127
N1—H3*N*1⋯O7^iv^	0.89	1.88	2.7645 (15)	169
N2—H1*N*2⋯O3^ii^	0.89	1.94	2.7956 (15)	160
N2—H2*N*2⋯O2	0.89	1.98	2.8637 (15)	169
N2—H3*N*2⋯O6^iii^	0.89	1.99	2.8053 (14)	152
C1—H1*B*⋯O5^ii^	0.97	2.52	3.3333 (19)	141
C8—H8*B*⋯O7	0.97	2.40	3.1558 (17)	135

Symmetry codes: (i) 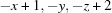; (ii) 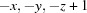; (iii) 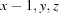; (iv) 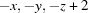.

**Fig. 1.**
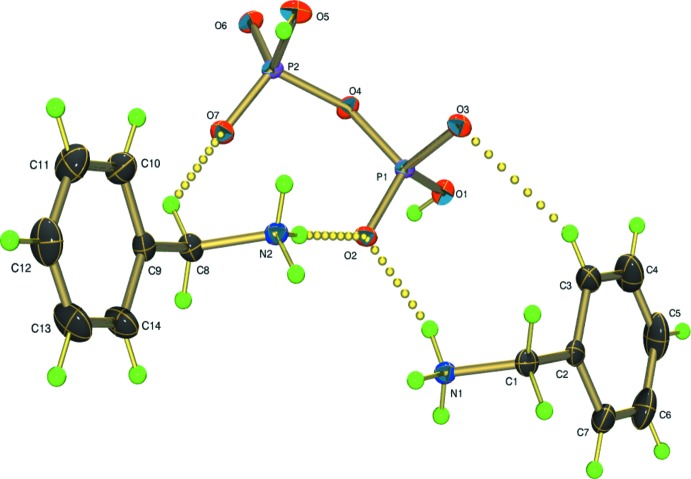
An ORTEP (Farrugia, 2012) view of (I) with displacement ellipsoids are drawn at the 30% probability level. H atoms are represented as small spheres of arbitrary radii. Hydrogen bonds are represented as dashed lines.

**Fig. 2.**
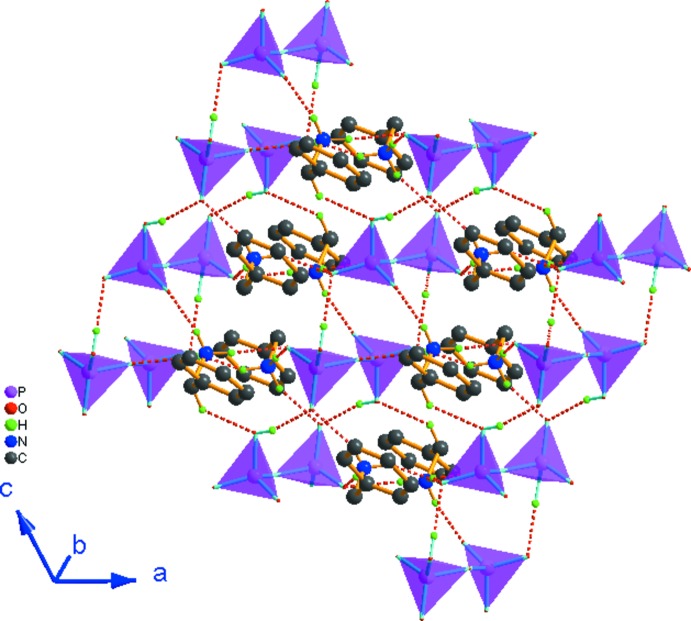
Part of the crystal structure of (I) with hydrogen bonds represented as red dashed lines. The H-atoms not involved in H-bonds are omitted.

## supplementary crystallographic information

### 1. Comment

There is current interest in the chemistry of diphosphate materials. They are involved in a variety of bioenergetic (Ernester, 1992; Lipscomb & Strater, 1996) and catalytic processes (Centi *et al.*, 1988; Chen *et al.*, 2002; Ballarini *et al.*, 2006). Considering their relevance in several application areas, we are interested in this type of anion in building new hybrid materials associated to organic cations. We report here, the synthesis and the crystal structure of the title compound (I).

The asymmetric unit of (I) shown in Fig. 1, contains one diphosphate [H_2_P_2_O_7_]^2-^ anion and two crystallographically independent benzylammonium cations. The two PO_4_ tetrahedral groups are bridged *via* the O4 bridging oxygen atom with P1—O4—P2 = 133.33 (6)° so as to form the diphosphate anion with a bent configurtation. The conformation is eclipsed evidenced by the psuedo-torsion angle O3—P1···P2—O7 = -6.9°. In the diphosphate group, the longest P—O distances correspond to the bridging oxygen atom with average value d(P—O4) = 1.6104 (8) Å, the intermediate distances are the P—OH bonding [*d*(P1—O1) = 1.5693 (9) Å, d(P2—O5) = 1.5607 (10) Å], whereas the shortest distances, ranging between 1.4762 (9) Å and 1.4987 (8) Å are related to the terminal oxygen atoms. The average value of the O—P—O angles is 109.25 (5)°. These geometrical features are in same magnitude as observed for diphosphate groups (Akriche *et al.*, 2005; Ahmed *et al.*, 2006; Akriche *et al.*, 2008; Elboulali *et al.*, 2013).

In the crystal, O—H···O and N—H···O hydrogen bonds link the cations and anions forming a two-dimensional network parallel to (010) (Table 1 and Fig. 2). Within this network, weak C—H···O hydrogen bonds are observed (Desiraju, 1991; Steiner 2002).

### 2. Experimental

Prismatic single crystals of the title compound were prepared at room temperature by slow evaporation of a mixture of an aqueous solution (20 ml) of diphosphoric acid (5 mmol) and an ethanolic solution (10 ml) of benzylamine (4 mmol, 0.44 ml). The diphosphoric acid was produced from Na_4_P_2_O_7_ by using a cation-exchange resin (Amberlite IR 120).

### 3. Refinement

All H atoms were placed in calculated positions and treated as riding, with C—H = 0.93 and 0.97 Å respectively for benzene rings and CH_2_ groups, N—H = 0.89 Å and O—H = 0.82 Å with *U*_iso_(H) = 1.2*U*_eq_(C) or *U*_iso_(H) = 1.5*U*_eq_(N,*O*).

### Crystal data

**Table:**

2C_7_H_10_N^+^·H_2_P_2_O_7_^2^^−^	*F*(000) = 824
*M**_r_* = 392.27	*D*_x_ = 1.426 Mg m^−^^3^
Monoclinic, *P*2_1_/*c*	Mo *K*α radiation, λ = 0.71073 Å
*a* = 8.1337 (2) Å	Cell parameters from 25 reflections
*b* = 28.9015 (9) Å	θ = 9–11°
*c* = 8.4727 (2) Å	µ = 0.28 mm^−^^1^
β = 113.449 (1)°	*T* = 293 K
*V* = 1827.24 (9) Å^3^	Prism, colourless
*Z* = 4	0.3 × 0.2 × 0.1 mm

### Data collection

**Table:**

Nonius KappaCCD diffractometer	5946 reflections with *I* > 2σ(*I*)
Radiation source: fine-focus sealed tube	*R*_int_ = 0.025
Detector resolution: 9 pixels mm^-1^	θ_max_ = 34.3°, θ_min_ = 2.7°
CCD rotation images, thick slices scans	*h* = −12→11
27919 measured reflections	*k* = −45→45
7410 independent reflections	*l* = −13→12

### Refinement

**Table:**

Refinement on *F*^2^	0 restraints
Least-squares matrix: full	Hydrogen site location: inferred from neighbouring sites
*R*[*F*^2^ > 2σ(*F*^2^)] = 0.043	H-atom parameters constrained
*wR*(*F*^2^) = 0.127	*w* = 1/[σ^2^(*F*_o_^2^) + (0.0651*P*)^2^ + 0.3854*P*] where *P* = (*F*_o_^2^ + 2*F*_c_^2^)/3
*S* = 1.06	(Δ/σ)_max_ = 0.001
7410 reflections	Δρ_max_ = 0.52 e Å^−^^3^
230 parameters	Δρ_min_ = −0.28 e Å^−^^3^

### Special details

**Table:**

Geometry. All e.s.d.'s (except the e.s.d. in the dihedral angle between two l.s. planes) are estimated using the full covariance matrix. The cell e.s.d.'s are taken into account individually in the estimation of e.s.d.'s in distances, angles and torsion angles; correlations between e.s.d.'s in cell parameters are only used when they are defined by crystal symmetry. An approximate (isotropic) treatment of cell e.s.d.'s is used for estimating e.s.d.'s involving l.s. planes.

### Fractional atomic coordinates and isotropic or equivalent isotropic displacement parameters (Å^2^)

**Table:**

	*x*	*y*	*z*	*U*_iso_*/*U*_eq_	
P1	0.16846 (4)	0.04396 (2)	0.78619 (4)	0.02353 (7)	
P2	0.35638 (4)	−0.03781 (2)	0.72893 (4)	0.02459 (8)	
O1	0.27096 (13)	0.08328 (3)	0.91571 (12)	0.0351 (2)	
H1O1	0.3218	0.0723	1.0123	0.053*	
O2	0.05569 (11)	0.01565 (3)	0.85084 (12)	0.03180 (19)	
O3	0.07455 (11)	0.06475 (3)	0.61148 (11)	0.03265 (19)	
O4	0.33579 (11)	0.01439 (3)	0.78719 (13)	0.03276 (19)	
O5	0.25728 (13)	−0.03691 (4)	0.52889 (12)	0.0401 (2)	
H5O5	0.1642	−0.0519	0.4998	0.060*	
O6	0.55388 (11)	−0.04139 (3)	0.77466 (12)	0.0332 (2)	
O7	0.27351 (13)	−0.07023 (4)	0.81039 (13)	0.0367 (2)	
N1	−0.27361 (14)	0.05080 (4)	0.87008 (14)	0.0304 (2)	
H1N1	−0.1667	0.0467	0.8665	0.046*	
H2N1	−0.3407	0.0258	0.8287	0.046*	
H3N1	−0.2600	0.0554	0.9784	0.046*	
N2	−0.16351 (14)	−0.06105 (4)	0.67481 (15)	0.0320 (2)	
H1N2	−0.1638	−0.0614	0.5697	0.048*	
H2N2	−0.0859	−0.0399	0.7386	0.048*	
H3N2	−0.2727	−0.0541	0.6681	0.048*	
C1	−0.36307 (19)	0.09184 (5)	0.76385 (18)	0.0384 (3)	
H1A	−0.4815	0.0955	0.7640	0.046*	
H1B	−0.3769	0.0869	0.6460	0.046*	
C2	−0.25602 (19)	0.13517 (5)	0.83218 (19)	0.0372 (3)	
C3	−0.1281 (3)	0.14896 (6)	0.7728 (3)	0.0544 (4)	
H3	−0.1092	0.1318	0.6887	0.065*	
C4	−0.0272 (3)	0.18878 (8)	0.8397 (4)	0.0820 (8)	
H4	0.0598	0.1978	0.8008	0.098*	
C5	−0.0551 (4)	0.21432 (8)	0.9606 (4)	0.0865 (8)	
H5	0.0111	0.2411	1.0026	0.104*	
C6	−0.1804 (3)	0.20080 (7)	1.0213 (3)	0.0717 (6)	
H6	−0.1980	0.2183	1.1055	0.086*	
C7	−0.2814 (2)	0.16112 (6)	0.9578 (2)	0.0498 (4)	
H7	−0.3662	0.1520	0.9996	0.060*	
C8	−0.11012 (18)	−0.10755 (5)	0.75535 (19)	0.0381 (3)	
H8A	−0.1077	−0.1069	0.8707	0.046*	
H8B	0.0098	−0.1148	0.7643	0.046*	
C9	−0.23714 (18)	−0.14468 (5)	0.65266 (18)	0.0367 (3)	
C10	−0.2192 (2)	−0.16351 (7)	0.5112 (2)	0.0522 (4)	
H10	−0.1301	−0.1526	0.4781	0.063*	
C11	−0.3324 (3)	−0.19860 (8)	0.4172 (3)	0.0655 (5)	
H11	−0.3191	−0.2111	0.3219	0.079*	
C12	−0.4640 (3)	−0.21471 (7)	0.4657 (3)	0.0689 (6)	
H12	−0.5382	−0.2388	0.4050	0.083*	
C13	−0.4863 (3)	−0.19547 (8)	0.6032 (3)	0.0708 (6)	
H13	−0.5781	−0.2058	0.6334	0.085*	
C14	−0.3720 (3)	−0.16037 (7)	0.6985 (3)	0.0563 (4)	
H14	−0.3868	−0.1476	0.7928	0.068*	

### Atomic displacement parameters (Å^2^)

**Table:**

	*U*^11^	*U*^22^	*U*^33^	*U*^12^	*U*^13^	*U*^23^
P1	0.01799 (12)	0.03007 (15)	0.02214 (13)	0.00091 (9)	0.00756 (9)	0.00035 (10)
P2	0.01780 (12)	0.03298 (16)	0.02239 (13)	0.00009 (9)	0.00736 (9)	−0.00164 (10)
O1	0.0328 (4)	0.0354 (5)	0.0301 (4)	0.0006 (4)	0.0051 (3)	−0.0056 (3)
O2	0.0255 (4)	0.0393 (5)	0.0350 (4)	0.0009 (3)	0.0167 (3)	0.0055 (4)
O3	0.0284 (4)	0.0423 (5)	0.0243 (4)	0.0000 (3)	0.0073 (3)	0.0042 (3)
O4	0.0219 (4)	0.0333 (5)	0.0454 (5)	−0.0004 (3)	0.0159 (3)	−0.0050 (4)
O5	0.0276 (4)	0.0650 (7)	0.0250 (4)	−0.0070 (4)	0.0076 (3)	−0.0027 (4)
O6	0.0191 (3)	0.0444 (5)	0.0345 (4)	0.0029 (3)	0.0089 (3)	−0.0056 (4)
O7	0.0382 (5)	0.0374 (5)	0.0379 (5)	−0.0049 (4)	0.0187 (4)	0.0000 (4)
N1	0.0269 (4)	0.0310 (5)	0.0351 (5)	−0.0003 (4)	0.0143 (4)	−0.0008 (4)
N2	0.0294 (5)	0.0337 (6)	0.0367 (5)	−0.0035 (4)	0.0174 (4)	−0.0033 (4)
C1	0.0359 (6)	0.0401 (7)	0.0359 (6)	0.0054 (5)	0.0109 (5)	0.0047 (5)
C2	0.0370 (6)	0.0323 (6)	0.0423 (7)	0.0083 (5)	0.0158 (5)	0.0087 (5)
C3	0.0575 (9)	0.0460 (9)	0.0719 (11)	0.0067 (7)	0.0387 (9)	0.0124 (8)
C4	0.0679 (13)	0.0560 (13)	0.132 (2)	−0.0072 (10)	0.0504 (15)	0.0200 (14)
C5	0.0729 (14)	0.0381 (10)	0.127 (2)	−0.0086 (10)	0.0175 (15)	0.0029 (12)
C6	0.0788 (14)	0.0419 (10)	0.0772 (14)	0.0134 (9)	0.0128 (11)	−0.0112 (9)
C7	0.0545 (9)	0.0426 (8)	0.0528 (9)	0.0113 (7)	0.0217 (7)	0.0000 (7)
C8	0.0315 (6)	0.0387 (7)	0.0395 (6)	−0.0033 (5)	0.0093 (5)	0.0023 (5)
C9	0.0324 (6)	0.0309 (6)	0.0426 (7)	−0.0009 (5)	0.0106 (5)	0.0053 (5)
C10	0.0475 (8)	0.0508 (9)	0.0612 (10)	−0.0059 (7)	0.0247 (8)	−0.0099 (8)
C11	0.0687 (12)	0.0545 (11)	0.0703 (12)	−0.0080 (9)	0.0244 (10)	−0.0220 (9)
C12	0.0640 (12)	0.0480 (10)	0.0782 (14)	−0.0195 (9)	0.0110 (10)	−0.0093 (10)
C13	0.0656 (12)	0.0685 (13)	0.0795 (14)	−0.0334 (10)	0.0301 (11)	−0.0005 (11)
C14	0.0568 (10)	0.0588 (11)	0.0584 (10)	−0.0203 (8)	0.0283 (8)	−0.0006 (8)

### Geometric parameters (Å, º)

**Table:**

P1—O2	1.4869 (9)	C3—C4	1.396 (3)
P1—O3	1.4953 (9)	C3—H3	0.9300
P1—O1	1.5693 (9)	C4—C5	1.353 (4)
P1—O4	1.6042 (9)	C4—H4	0.9300
P2—O7	1.4762 (10)	C5—C6	1.369 (4)
P2—O6	1.4987 (9)	C5—H5	0.9300
P2—O5	1.5607 (10)	C6—C7	1.388 (3)
P2—O4	1.6166 (10)	C6—H6	0.9300
O1—H1O1	0.8200	C7—H7	0.9300
O5—H5O5	0.8200	C8—C9	1.5035 (19)
N1—C1	1.4917 (17)	C8—H8A	0.9700
N1—H1N1	0.8900	C8—H8B	0.9700
N1—H2N1	0.8900	C9—C10	1.376 (2)
N1—H3N1	0.8900	C9—C14	1.378 (2)
N2—C8	1.4914 (18)	C10—C11	1.388 (3)
N2—H1N2	0.8900	C10—H10	0.9300
N2—H2N2	0.8900	C11—C12	1.372 (3)
N2—H3N2	0.8900	C11—H11	0.9300
C1—C2	1.504 (2)	C12—C13	1.366 (3)
C1—H1A	0.9700	C12—H12	0.9300
C1—H1B	0.9700	C13—C14	1.396 (3)
C2—C3	1.382 (2)	C13—H13	0.9300
C2—C7	1.383 (2)	C14—H14	0.9300
			
O2—P1—O3	116.03 (5)	C4—C3—H3	120.1
O2—P1—O1	112.01 (6)	C5—C4—C3	120.6 (2)
O3—P1—O1	108.73 (5)	C5—C4—H4	119.7
O2—P1—O4	110.57 (5)	C3—C4—H4	119.7
O3—P1—O4	108.51 (5)	C4—C5—C6	120.2 (2)
O1—P1—O4	99.70 (5)	C4—C5—H5	119.9
O7—P2—O6	118.50 (6)	C6—C5—H5	119.9
O7—P2—O5	112.56 (6)	C5—C6—C7	120.3 (2)
O6—P2—O5	108.61 (5)	C5—C6—H6	119.9
O7—P2—O4	109.12 (5)	C7—C6—H6	119.9
O6—P2—O4	102.49 (5)	C2—C7—C6	119.97 (19)
O5—P2—O4	104.16 (6)	C2—C7—H7	120.0
P1—O1—H1O1	109.5	C6—C7—H7	120.0
P1—O4—P2	133.33 (6)	N2—C8—C9	111.74 (11)
P2—O5—H5O5	109.5	N2—C8—H8A	109.3
C1—N1—H1N1	109.5	C9—C8—H8A	109.3
C1—N1—H2N1	109.5	N2—C8—H8B	109.3
H1N1—N1—H2N1	109.5	C9—C8—H8B	109.3
C1—N1—H3N1	109.5	H8A—C8—H8B	107.9
H1N1—N1—H3N1	109.5	C10—C9—C14	119.20 (15)
H2N1—N1—H3N1	109.5	C10—C9—C8	119.97 (14)
C8—N2—H1N2	109.5	C14—C9—C8	120.83 (15)
C8—N2—H2N2	109.5	C9—C10—C11	120.81 (18)
H1N2—N2—H2N2	109.5	C9—C10—H10	119.6
C8—N2—H3N2	109.5	C11—C10—H10	119.6
H1N2—N2—H3N2	109.5	C12—C11—C10	119.7 (2)
H2N2—N2—H3N2	109.5	C12—C11—H11	120.2
N1—C1—C2	111.18 (11)	C10—C11—H11	120.2
N1—C1—H1A	109.4	C13—C12—C11	120.08 (18)
C2—C1—H1A	109.4	C13—C12—H12	120.0
N1—C1—H1B	109.4	C11—C12—H12	120.0
C2—C1—H1B	109.4	C12—C13—C14	120.38 (19)
H1A—C1—H1B	108.0	C12—C13—H13	119.8
C3—C2—C7	119.23 (16)	C14—C13—H13	119.8
C3—C2—C1	120.29 (15)	C9—C14—C13	119.82 (19)
C7—C2—C1	120.46 (14)	C9—C14—H14	120.1
C2—C3—C4	119.7 (2)	C13—C14—H14	120.1
C2—C3—H3	120.1		
			
O2—P1—O4—P2	44.21 (10)	C3—C2—C7—C6	0.7 (2)
O3—P1—O4—P2	−84.10 (10)	C1—C2—C7—C6	179.28 (15)
O1—P1—O4—P2	162.27 (9)	C5—C6—C7—C2	−0.2 (3)
O7—P2—O4—P1	−51.43 (10)	N2—C8—C9—C10	81.81 (18)
O6—P2—O4—P1	−177.87 (9)	N2—C8—C9—C14	−98.38 (17)
O5—P2—O4—P1	68.98 (10)	C14—C9—C10—C11	−1.4 (3)
N1—C1—C2—C3	90.66 (17)	C8—C9—C10—C11	178.43 (17)
N1—C1—C2—C7	−87.87 (16)	C9—C10—C11—C12	0.1 (3)
C7—C2—C3—C4	−0.2 (3)	C10—C11—C12—C13	1.7 (4)
C1—C2—C3—C4	−178.79 (18)	C11—C12—C13—C14	−2.0 (4)
C2—C3—C4—C5	−0.8 (4)	C10—C9—C14—C13	1.0 (3)
C3—C4—C5—C6	1.3 (4)	C8—C9—C14—C13	−178.81 (18)
C4—C5—C6—C7	−0.8 (4)	C12—C13—C14—C9	0.7 (4)

### Hydrogen-bond geometry (Å, º)

**Table:**

*D*—H···*A*	*D*—H	H···*A*	*D*···*A*	*D*—H···*A*
O1—H1*O*1···O6^i^	0.82	1.90	2.7208 (13)	174
O5—H5*O*5···O3^ii^	0.82	1.83	2.6061 (13)	158
N1—H1*N*1···O2	0.89	2.07	2.9292 (13)	162
N1—H2*N*1···O6^iii^	0.89	2.10	2.9698 (15)	166
N1—H2*N*1···O4^iii^	0.89	2.53	3.1493 (13)	127
N1—H3*N*1···O7^iv^	0.89	1.88	2.7645 (15)	169
N2—H1*N*2···O3^ii^	0.89	1.94	2.7956 (15)	160
N2—H2*N*2···O2	0.89	1.98	2.8637 (15)	169
N2—H3*N*2···O6^iii^	0.89	1.99	2.8053 (14)	152
C1—H1*B*···O5^ii^	0.97	2.52	3.3333 (19)	141
C8—H8*B*···O7	0.97	2.40	3.1558 (17)	135

Symmetry codes: (i) −*x*+1, −*y*, −*z*+2; (ii) −*x*, −*y*, −*z*+1; (iii) *x*−1, *y*, *z*; (iv) −*x*, −*y*, −*z*+2.

## References

[bb1] Ahmed, S., Samah, A. & Mohamed, R. (2006). *Acta Cryst.* E**62**, m1796–m1798.

[bb2] Akriche, S. & Rzaigui, M. (2005). *Acta Cryst.* E**61**, o2607–o2609.

[bb3] Akriche, S. & Rzaigui, M. (2008). *Struct. Chem.* **19**, 827–831.

[bb4] Ballarini, N., Cavani, F., Cortelli, C., Ligi, S., Pierelli, F., Trifiro, F., Fumagalli, C., Mazzoni, G. & Monti, T. (2006). *Top. Catal.* **38**, 147–156.

[bb5] Brandenburg, K. & Putz, H. (2005). *DIAMOND* Crystal Impact GbR, Bonn, Germany.

[bb6] Centi, G., Trifirò, F., Ebner, J. R. & Franchetti, V. M. (1988). *Chem. Rev.* **88**, 55–80.

[bb7] Chen, B. & Munson, E. J. (2002). *J. Am. Chem. Soc.* **124**, 1638–1652. 10.1021/ja010285v 11853438

[bb8] Desiraju, G. R. (1991). *Acc. Chem. Res.* **24**, 290–296.

[bb9] Duisenberg, A. J. M., Hooft, R. W. W., Schreurs, A. M. M. & Kroon, J. (2000). *J. Appl. Cryst.* **33**, 893–898.

[bb10] Duisenberg, A. J. M., Kroon-Batenburg, L. M. J. & Schreurs, A. M. M. (2003). *J. Appl. Cryst.* **36**, 220–229.

[bb11] Elboulali, A., Akriche, S., Al-Deyab, S. S. & Rzaigui, M. (2013). *Acta Cryst.* E**69**, o213–o214. 10.1107/S1600536812051616 PMC3569749 23424495

[bb12] Ernester, L. (1992). In *Molecular Mechanism in Bioenergetics* Amsterdam: Elsevier.

[bb13] Farrugia, L. J. (2012). *J. Appl. Cryst.* **45**, 849–854.

[bb14] Hooft, R. W. W. (1998). *COLLECT* Nonius BV, Delft, The Netherlands.

[bb15] Lipscomb, W. N. & Strater, N. (1996). *Chem. Rev.* **96**, 2375–2433. 10.1021/cr950042j 11848831

[bb16] Sheldrick, G. M. (2008). *Acta Cryst.* A**64**, 112–122. 10.1107/S0108767307043930 18156677

[bb17] Steiner, T. (2002). *Angew. Chem. Int. Ed.* **41**, 48–76.

